# Association between Systemic Immune Inflammation Index and Cognitive Impairment after Acute Ischemic Stroke

**DOI:** 10.3390/brainsci13030464

**Published:** 2023-03-09

**Authors:** Yuanfei Bao, Lingling Wang, Chaopin Du, Yan Ji, Yiwei Dai, Wei Jiang

**Affiliations:** 1 Department of Neurology, Nantong Third People’s Hospital, Nantong University, Nantong 226000, China; 2 Clinical Laboratory, Nantong First People’s Hospital, Nantong 226000, China; 3 Department of Neurology, Wuxi No. 2 People’s Hospital, Jiangnan University Medical Center, Wuxi 214000, China

**Keywords:** systemic immune-inflammation, cognitive impairment, ischemic stroke, prognosis, MoCA

## Abstract

Background and Aims: Post-stroke cognitive impairment (PSCI) is one of the major complications after ischemic stroke. PSCI has been shown to be associated with low-grade systemic inflammation. As a novel inflammatory marker, the systemic immune-inflammation (SII) index could reflect clinical outcomes in severe cardiovascular diseases. We therefore performed a prospective study to investigate the correlation between the SII index and the risk of PSCI in patients with ischemic stroke. Methods: We prospectively enrolled 254 patients with ischemic stroke with symptoms onset <72 h. The SII index was detected within 24 h after admission. The Montreal Cognitive Scale (MoCA) was utilized to evaluate cognitive function, and PSCI was defined as a MoCA score of <25 points. Results: During the 3-month follow-up, 70 participants (27.6%) had mild cognitive impairment and 60 (23.6%) had severe cognitive impairment. In binary logistic regression analysis, each one-standard deviation increase in the SII index was significantly associated with the prevalence of PSCI after adjusting for age, sex, and other confounders (odds ratio 2.341; 95% confidence interval, 1.439–3.809, *p* = 0.001). Similar significant findings were observed when SII was defined as a categorical variable. In addition, the multiple-adjusted spline regression model showed a linear association between the SII index and cognitive impairment (*p* = 0.003 for linearity). Conclusions: Our study indicated that an increased SII index was closely related to PSCI at 3 months in patients with ischemic stroke. Further research is required to evaluate the efficacy of inflammation management in these patients.

## 1. Introduction

Stroke is one of the diseases with the highest disability and morbidity in China and worldwide [1,2,3,4,5]. Post-stroke cognitive impairment (PSCI) is a frequent complication of ischemic stroke, and it affects 20–80% of patients, depending on diagnostic criteria, countries, and races [6,7,8]. The period between stroke onset and the presence of PSCI can be recognized as a treatment window for early intervention to protect cognitive function [9]. Therefore, determining reliable predictors for PSCI is of vital importance for continuously improving the prognosis of stroke.

In recent years, several researchers have progressively recognized the secondary injury of the brain’s inflammatory response after ischemic stroke [10,11]. Preclinical and clinical studies have confirmed a causal link between sterile low-grade inflammation and the pathogenesis of stroke [12,13,14]. Accumulating evidence also reported that several pro-inflammatory and inflammatory molecules were involved in the inflammatory response to cognitive impairment, including C-reactive protein, interleukin-6, and interleukin-10 [15]. The crosstalk between the immune system and the central nervous system during ischemic stroke may be the underlying mechanism of brain tissue injury and complications. The systemic immune-inflammation index (SII) is a new type of comprehensive inflammation index combining peripheral lymphocytes, neutrophils, and platelet counts [16]. The SII was first proposed in a variety of cancer research, aiming to identify patients with a high risk of recurrence or death [16,17,18]. Zhang et al. performed a meta-analysis and reported that increased pretreatment SII significantly correlated with worse overall survival and recurrence-free survival/progression-free survival in biliary tract cancers [18]. SII can be used to comprehensively evaluate the inflammation status better than the neutrophil-to-lymphocyte ratio, lymphocyte-to-monocyte ratio, platelet-to-lymphocyte ratio, and platelet volume. In the central nervous system, the SII index could differentiate the high- and low-grade gliomas [19]. In recent years, the SII score has been studied as a marker for stroke outcomes [20,21,22,23]. Weng et al. conducted a retrospective study consecutively including 365 patients who were treated with intravenous thrombolysis, which found that SII is correlated with stroke severity and can be a novel prognostic biomarker [22]. Yi et al. retrospectively reviewed prospectively collected data from the stroke database of each institution and found that decreased SII index was associated with favorable clinical outcomes in patients who underwent mechanical thrombectomy for large artery occlusion [23]. However, the results of previous studies on ischemic stroke did not sufficiently demonstrate the effectiveness of the SII score as a potential predictor of PSCI after ischemic stroke. In our prospective cohort study, we aimed to systematically evaluate the relationship between the SII index and the risk of PSCI in patients with acute ischemic stroke.

## 2. Materials and Methods

### 2.1. Study Sample

First-ever patients with ischemic stroke who were treated at the Nantong Third People’s Hospital between September 2021 and October 2022 were prospectively selected. Patients were enrolled based on the following criterion: (1) age ≥ 18 years; (2) had time from symptom onset to admission within 72 h; (3) had a baseline National Institutes of Health Stroke Scale (NIHSS) score ≤ 8 points. The exclusion criteria were as follows: (1) patients with obvious disorder of consciousness; (2) patients with obvious apraxia and/or aphasia who were unable to complete the questionnaire; (3) patients complicated with diseases that might affect cognitive function, such as brain tumor, Parkinson’s syndrome, Alzheimer’s disease, frontotemporal dementia, schizophrenia, major depression, and alcohol abuse; (4) patients complicated with diseases which might affect inflammatory conditions, such as malignant tumors, acute infection, and trauma. This study was reviewed and approved by the Medical Research Ethics Committee of the Nantong Third People’s Hospital (MB2020034). The written informed consent for participation in the study was obtained from each participant or their caregivers.

### 2.2. Baseline Data Collection

Demographic characteristics, vascular risk factors, clinical data, and laboratory data were all collected after admission. The blood pressure was measured after admission. Hypertension was defined as a history of hypertension and/or the use of antihypertensive medications. Hyperlipidemia was defined as a history of hyperlipidemia and/or having received treatment for dyslipidemia. Diabetes mellitus was defined as a history of diabetes and/or the use of glucose-lowering agents. The baseline stroke severity was assessed by experienced neurologists using the NIHSS [24]. The stroke subtype was classified according to the criteria of Trial of Org 10172 in Acute Stroke Treatment [25]. The laboratory data, including peripheral blood count, total cholesterol, triglyceride, high-density lipoprotein, low-density lipoprotein, fasting blood glucose, high-sensitivity C-reactive protein (Hs-CRP), and SII index, were recorded. Blood samples were routinely obtained within 24 h of hospital admission. The lipid profile and fasting blood glucose were detected by Chemistry Analyzer (AU480, Beckman Coulter, Brea, CA, USA). Then, the cell counts were analyzed by an auto-analyzer (XE-2100, Sysmex, Kobe, Japan) and utilized to calculate the SII index. According to previous studies [21,26,27], the SII index was calculated as: platelet count × neutrophil count/lymphocyte count.

### 2.3. Assessment of Cognitive Function

The study outcome was PSCI at 3 months after stroke, assessed by a professionally trained neurologist who was blinded to the clinical and laboratory data, using the Montreal Cognitive Assessment (MoCA) scale [28]. The total MoCA score is 30 points. To correct for errors, 1 point was added to the total MoCA score in participants with <12 years of education. In this analysis, a score of <25 on the total MoCA indicated the presence of PSCI [29,30]. Furthermore, according to the recommended cutoffs, the degree of cognitive impairment was categorized as follows: severe cognitive impairment (0–19), mild cognitive impairment (20–24), and no cognitive impairment (25–30).

### 2.4. Statistical Analysis

Statistical analyses were performed using the statistical software SPSS (version 25.0; IBM, New York, NY, USA), and R (version 4.0.1; The R Project for Statistical Computing, Vienna, Austria). Continuous variables were expressed as means with standard deviation (SD) or median with interquartile range (IQR). Categorical variables were described by frequencies with percentages. Differences in baseline characteristics in the patients with and without were compared using an independent sample t-test, an χ^2^ test, one-way analysis of variance (Bonferroni post hoc test), and a Kruskal–Wallis H-test. Logistic regression analysis was utilized to determine the association of the SII index with the presence and severity of PSCI after adjusting for potential confounders. All multivariate analyses were first adjusted for age and gender (Model 1) and additionally adjusted for all variables (including age, gender, education years, hypertension, diabetes mellitus, stroke subtypes, baseline stroke severity, and Hs-CRP levels; Model 2). Results were shown as adjusted odds ratio (OR) (95% confidence interval, CI).

Furthermore, we performed the restricted cubic splines with 3 knots (at the 5th, 50th, and 95th percentiles) to explore the pattern and magnitude of the relationship between the SII index and clinical outcomes [31]. A *p-*value < 0.05 was considered statistically significant for all the analyses.

## 3. Results

### 3.1. Baseline Characteristics

In this study, we identified 254 patients with ischemic stroke who met our inclusion criteria. The eligible participants included 143 men and 111 women with a mean age of 65.5 ± 10.2 years. Among these patients with ischemic stroke, 68.5% had hypertension, 27.6% had diabetes mellitus, and 15.0% had hyperlipidemia. The median (IQR) SII index was 574.2 (337.6–888.9) × 10^9^/L, and the median (IQR) NIHSS score at admission was 3.0 (2.0–5.0) points. The demographic and clinical characteristics of the study population stratified by the SII quartiles are presented in Table 1. Age, diastolic blood pressure, the prevalence of PSCI, and Hs-CRP levels differed significantly with the increasing quartile of the SII index. However, sex and the presence of vascular risk factors did not differ significantly among the categories.

### 3.2. Prevalence and Risk Factors of PSCI

During the 3-month follow-up, 130 participants (51.2%) had PSCI. Table 2 demonstrated the data of the comparison between patients with and without PSCI. On univariate analysis, patients with PSCI were older (mean, 67.3 ± 8.9 years versus 63.4 ± 11.1 years; *p* = 0.004), had a higher prevalence of hypertension (74.6% versus 62.1%; *p* = 0.032), diabetes mellitus (33.8% versus 21.0%; *p* = 0.022), and large artery atherosclerosis stroke (*p* = 0.035), and had a higher baseline NIHSS score (median, 4.0 score versus 3.0 score; *p* = 0.001), Hs-CRP levels (median, 6.8 mg/L versus 4.5 mg/L; *p* = 0.031), and SII index (median, 653.9 × 10^9^/L versus 493.1 × 10^9^/L; *p* = 0.001). Furthermore, educational years were lower in patients with PSCI than in patients without PSCI (median, 9.0 years versus 9.0 years; *p* = 0.001).

### 3.3. Logistic Regression Analysis for the Relationship between the SII Index and PSCI

**Figure 1:**
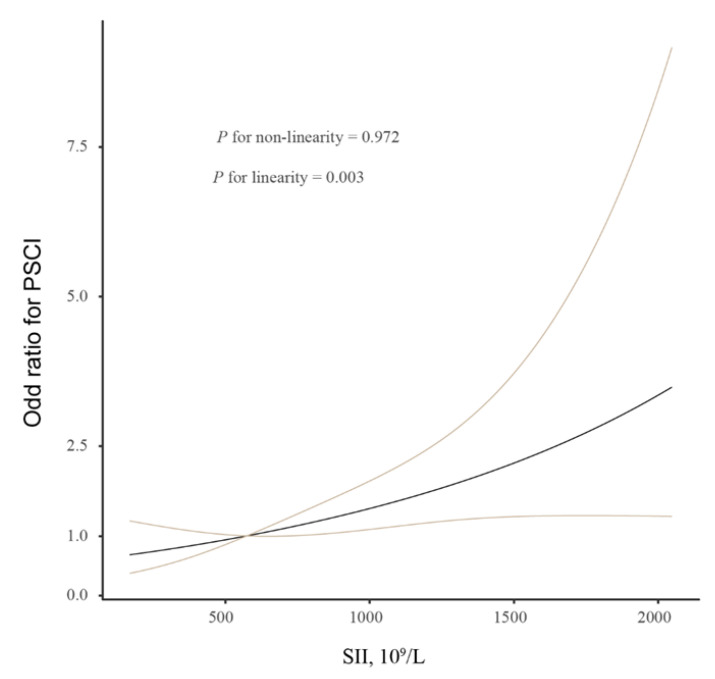
Restricted cubic spline evaluated the association between SII index and PSCI. Association was fitted with restricted cubic spline with 3 knots (at 5th, 50th, and 95th percentiles) controlling for demographic characteristics, education years, hypertension, diabetes, stroke subtypes, baseline NIHSS score, and Hs-CRP levels. The midpoint of SII index was set as the reference point. Abbreviations: PSCI, post-stroke cognitive impairment; SII, systemic immune-inflammation.

In order to study whether the SII index could be used as an effective variable for the diagnosis of the presence and severity of PSCI, we utilized a multivariate logistic regression analysis. The results of the logistic analysis were demonstrated in Table 3. After controlling for age and sex, a high SII index is a significant risk factor for PSCI (per 1-SD increase, OR, 2.045; 95% CI, 1.347–3.106; *p* = 0.001; Model 1). In Model 2, after further controlling for education years, hypertension, diabetes, stroke subtypes, baseline NIHSS score, and Hs-CRP levels, an increased SII index also significantly correlated with 3-month PSCI (per 1-SD increase, OR, 2.341; 95% CI, 1.439–3.809; *p* = 0.001). Similar significant findings were observed when SII was categorized according to the quartile. In addition, the multiple-adjusted spline regression model showed a linear association between the SII index and cognitive impairment (*p* = 0.003 for linearity; Figure 1).

Among these patients with PSCI, 70 participants (27.6%) had mild PSCI and 60 (23.6%) had severe PSCI. After controlling for the potential confounders, the ordinal regression analysis further confirmed the close association between the SII index and severity of PSCI (per SD increase, OR, 1.879; 95% CI, 1.324–2.537; *p* = 0.001).

## 4. Discussion

This study showed that patients with acute ischemic stroke suffering from cognitive impairment had a significantly higher SII index than those without cognitive impairment. Furthermore, even after adjusting for potential confounders, the positive association between the SII score and PSCI remained significant. SII, as a noninvasive and cost-effective serological inflammatory marker, represents potential prognostic predictors for PSCI screening and management.

Previously available studies on PSCI used inconsistent definitions and study populations, leading to discrepancies in incidence rates and associated factors. This study reported that 51.2% of patients who suffered from ischemic stroke present with PSCI at 3 months, which is consistent with earlier literature [6,7,8]. Our data further showed that the PSCI risk was higher in patients with older age and low education levels, which was similar to previous studies [6,32]. Elderly patients often accompany decreased cerebral blood flow and arteriosclerosis, which is more likely to cause cognitive dysfunction. Additionally, several studies have found that patients with a higher education level tend to have a larger cognitive reserve capacity after stroke [33]. We also confirmed that patients with diabetes are at higher risk for cognitive impairment. Diabetes mellitus is often accompanied by a more severe oxidative stress reaction and longer exposure to glycolipid metabolic disorders. These factors significantly mediate the correlation between diabetes and cognitive impairment [34].

The SII index was established by Hu and designed to predict the clinical outcomes in hepatocellular carcinoma patients after operative treatment [16], which showed that the predictive value of the SII index was more accurate than those indexes that use only one or two cell subtypes. In recent years, a higher level of the SII index is a predicting factor for poor outcomes in brain pathologies such as glioma, aneurysmal subarachnoid hemorrhage, ischemic stroke, and post-stroke depression [19,20,21,22,23,24,35]. Weng et al. conducted a retrospective study consecutively including 365 patients who were treated with intravenous thrombolysis, which found that SII is correlated with stroke severity and can be a novel prognostic biomarker [22]. Yi et al. retrospectively reviewed prospectively collected data from the stroke database of each institution and found that a decreased SII index was associated with favorable clinical outcomes in patients who underwent mechanical thrombectomy for large artery occlusion [23]. Additionally, Yun et al. have performed an analysis of inflammatory markers including SII in patients with aneurysmal subarachnoid hemorrhage and reported that an SII index value ≥960 × 10^9^/L was an independent predicting factor for poor prognosis [35]. In addition, data from a 2-year cross-sectional, stratified, multistage probability cluster survey demonstrated that the SII score was significantly higher in subjects with depression after ischemic stroke than those without it [20]. Our study extended the current knowledge about the adverse effect of a higher SII index in ischemic stroke, as it demonstrated a positive association between the SII score and the risk of PSCI at 3 months in patients with ischemic stroke.

Although PSCI is a heterogeneous condition after stroke, some researchers supposed that PSCI is a multisystem inflammatory disease [36]. The factors constituting the index might explain the effect of SII in PSCI. Neutrophils infiltrate into the ischemic tissue within several hours after stroke and peak at 48 h. Increased neutrophil levels lead to the increased expression of matrix metalloproteinase-9 and the release of pro-inflammatory mediators, which might damage the blood–brain barrier [37]. Furthermore, acute inflammatory secretions such as reactive oxygen species and proteolytic enzymes produced by neutrophils may further deteriorate these processes [38]. On the contrary, lymphocytes have been found to inhibit endothelial damage by regulating the inflammatory response [39]. The blood–brain barrier dysfunction may induce damage to the white matter and be related to a progression of cognitive impairment [40]. Platelets are an indicator of inflammation after stroke. Platelets interact directly with circulating leukocytes, thereby forming platelet–leukocyte aggregates and activating the innate immune response to ischemia [41]. In addition, the activation of platelets may have a negative effect on the phosphorylation and expression of brain-derived neurotrophic factor, which is a key molecule for memory in the healthy and the pathological brain [42]. Our observations, combined with results obtained in animal models and clinical studies, suggest that immuno-inflammatory responses may mediate cognitive function after ischemic stroke, offering a potential therapeutic target for intervention.

The strengths of our study include using a standardized research method, detailed psychological evaluation, and enrolling a homogeneous sample of patients with mild ischemic stroke, all of which make this group appropriate for examining the association between the SII score and the risk of PSCI. However, there are several limitations that should be addressed in this study. Firstly, the SII index used in this study was measured from one blood test only. It is useful to take routine blood tests constantly so as to detect the dynamic changes in SII score after ischemic stroke. Secondly, patients with obvious disorders of consciousness, apraxia and/or aphasia, and a history of cognitive decline were excluded from this study, which may underestimate the real PSCI rate and lead to a selection bias. Thirdly, the cross-sectional design of this study prevents causal inference. Further prospective studies with larger samples are needed to establish causality.

In conclusion, the current study revealed that the higher SII index at baseline was significantly associated with 3-month PSCI in patients with acute ischemic stroke. Further clinical research is warranted to examine whether managing inflammation reactions provides a potential preventive or therapeutic target for PSCI.

## Figures and Tables

**Table 1:** Baseline characteristics of the study sample stratified by SII index.

Variables	SII Index				
	First Quartile, *n* = 64	Second Quartile, *n* = 64	Third Quartile, *n* = 63	Fourth Quartile, *n* = 63	*p-*Value
Demographic characteristic					
Age, year	63.0 ± 9.8	65.7 ± 9.5	64.9 ± 11.9	68.6 ± 8.8	0.019
Male, %	41 (64.1)	28 (43.8)	40 (63.5)	34 (54.0)	0.108
Vascular risk factors, %					
Hypertension	48 (75.0)	42 (65.6)	40 (63.5)	44 (69.8)	0.516
Diabetes	20 (31.3)	18 (28.1)	20 (31.7)	12 (19.0)	0.350
Hyperlipidemia	7 (10.9)	10 (15.6)	9 (14.3)	12 (19.0)	0.640
Coronary heart disease	8 (12.5)	10 (15.6)	12 (19.0)	12 (19.0)	0.713
Current smoking	30 (46.9)	23 (35.9)	20 (31.7)	25 (39.7)	0.345
Current drinking	22 (34.4)	23 (35.9)	29 (46.0)	24 (38.1)	0.542
Clinical data					
Onset-to-blood drawing time, day	2.0 (1.0, 3.0)	2.0 (1.0, 3.0)	2.0 (1.0, 3.0)	2.0 (2.0, 3.0)	0.335
Educational status, years	9.0 (6.0, 12.0)	9.0 (6.0, 9.0)	8.0 (6.0, 9.0)	9.0 (7.0, 12.0)	0.341
Systolic blood pressure, mmHg	137.3 ± 17.0	139.7 ± 16.1	133.9 ± 18.4	137.1 ± 18.7	0.324
Diastolic blood pressure, mmHg	80.1 ± 8.6	81.9 ± 9.9	77.5 ± 10.0	83.7 ± 11.0	0.004
Baseline NIHSS, score	3.0 (2.0, 4.0)	3.0 (2.0, 4.0)	4.0 (2.0, 6.0)	4.0 (2.0, 5.0)	0.285
Previous antiplatelet, %	23 (35.9)	18 (28.1)	15 (23.8)	15 (23.8)	0.376
Previous statin, %	21 (32.8)	15 (23.4)	20 (31.7)	13 (20.6)	0.320
PSCI, %	28 (43.8)	25 (39.1)	30 (47.6)	47 (74.6)	0.001
Stroke subtypes, %					0.143
Large artery atherosclerosis	20 (31.3)	30 (46.9)	30 (47.6)	26 (41.3)	
Cardioembolism	20 (31.3)	11 (17.2)	12 (19.0)	10 (15.9)	
Small artery occlusion	23 (35.9)	20 (31.3)	16 (5.4)	20 (31.7)	
Others	1 (1.6)	3 (4.7)	5 (7.9)	7 (11.1)	
Laboratory data					
Total cholesterol, mmol/L	4.0 ± 1.1	3.9 ± 1.1	3.9 ± 0.9	4.0 ± 1.0	0.972
Triglyceride, mmol/L	1.5 ± 0.7	1.6 ± 0.7	1.5 ± 0.8	1.4 ± 0.6	0.434
Low-density lipoprotein, mmol/L	2.4 ± 0.8	2.4 ± 0.9	2.5 ± 0.6	2.5 ± 0.9	0.706
High-density lipoprotein, mmol/L	1.1 ± 0.3	1.1 ± 0.2	1.1 ± 0.3	1.1 ± 0.2	0.588
Hs-CRP, mg/L	4.8 (2.3, 8.1)	4.2 (2.2, 7.8)	5.8 (3.3, 9.1)	7.2 (3.3, 14.0)	0.049
Fasting blood glucose, mmol/L	6.3 ± 2.7	6.0 ± 2.5	6.5 ± 3.0	5.5 ± 2.5	0.174

Abbreviations: Hs-CRP, hyper-sensitive C-reactive protein; NIHSS, National Institutes of Health Stroke Scale; PSCI, post-stroke cognitive impairment; SII, systemic immune-inflation.

**Table 2:** Baseline data of the patients with and without PSCI.

Variables	With PSCI, *n* = 130	Without PSCI, *n* = 124	*p* Value
Demographic characteristic			
Age, year	67.3 ± 8.9	63.4 ± 11.1	0.004
Male, %	70 (53.8)	73 (58.9)	0.420
Vascular risk factors, %			
Hypertension	97 (74.6)	77 (62.1)	0.032
Diabetes	44 (33.8)	26 (21.0)	0.022
Hyperlipidemia	19 (14.6)	19 (15.3)	0.874
Coronary heart disease	22 (16.9)	20 (16.1)	0.865
Current smoking	48 (36.9)	50 (40.3)	0.578
Current drinking	49 (37.7)	49 (39.5)	0.765
Clinical data			
Onset-to-blood drawing time, day	2.0 (1.0, 3.0)	2.0 (1.0, 3.0)	0.812
Educational status, years	9.0 (6.0, 9.0)	9.0 (7.0, 12.0)	0.039
Systolic blood pressure, mmHg	136.7 ± 17.0	137.3 ± 18.3	0.806
Diastolic blood pressure, mmHg	81.1 ± 10.0	80.5 ± 10.0	0.637
Baseline NIHSS, score	4.0 (2.0, 6.0)	3.0 (2.0, 4.0)	0.001
Previous antiplatelet, %	38 (29.2)	33 (26.6)	0.642
Previous statin, %	37 (28.5)	32 (25.8)	0.634
Stroke subtypes, %			0.035
Large artery atherosclerosis	63 (48.5)	43 (34.7)	
Cardioembolism	28 (21.5)	25 (20.2)	
Small artery occlusion	30 (23.1)	49 (39.5)	
Others	9 (6.9)	7 (5.6)	
Laboratory data			
Total cholesterol, mmol/L	3.9 ± 1.0	4.0 ± 1.0	0.374
Triglyceride, mmol/L	1.5 ± 0.6	1.5 ± 0.8	0.968
Low-density lipoprotein, mmol/L	2.3 ± 0.8	2.5 ± 0.8	0.134
High-density lipoprotein, mmol/L	1.1 ± 0.3	1.1 ± 0.2	0.363
Hs-CRP, mg/L	6.8 (2.9, 10.0)	4.5 (2.6, 7.8)	0.031
Fasting blood glucose, mmol/L	6.1 ± 2.6	6.1 ± 2.8	0.983
SII index, ×10^9^/L	653.9 (352.7, 1117.9)	493.1 (300.5, 745.1)	0.001
SII quartile, %			0.001
First	28 (21.5)	36 (29.0)	
Second	25 (19.2)	39 (31.5)	
Third	30 (23.1)	33 (26.6)	
Fourth	47 (36.2)	16 (12.9)	

Abbreviations: Hs-CRP, hyper-sensitive C-reactive protein; NIHSS, National Institutes of Health Stroke Scale; PSCI, post-stroke cognitive impairment; SII, systemic immune-inflation.

**Table 3:** Multivariate analysis for the association of the SII index with PSCI.

Variables	PSCI		PSCI Severity	
	OR (95%CI)	*p* Value	OR (95%CI)	*p* Value
Crude model				
SII index (per-SD increase)	2.191 (1.437–3.338)	0.001	1.791 (1.301–2.480)	0.001
SII quartile				
First	Reference		Reference	
Second	0.820 (0.408–1.667)	0.590	2.630 (1.362–5.078)	0.004
Third	1.169 (0.581–2.351)	0.662	3.504 (1.787–6.862)	0.001
Fourth	3.777 (1.780–8.013)	0.001	2.669 (1.379–5.170)	0.003
Model 1				
SII index (per-SD increase)	2.045 (1.347–3.106)	0.001	1.726 (1.270–2.347)	0.001
SII quartile				
First	Reference		Reference	
Second	0.726 (0.352–1.496)	0.385	2.326 (1.189–4.549)	0.014
Third	1.106 (0.543–2.251)	0.781	3.340 (1.696–6.573)	0.001
Fourth	3.201 (1.486–6.894)	0.001	2.522 (1.291–4.918)	0.001
Model 2				
SII index (per-SD increase)	2.341 (1.439–3.809)	0.001	1.879 (1.324–2.537)	0.001
SII quartile				
First	Reference		Reference	
Second	0.644 (0.292–1.421)	0.276	2.927 (1.398–6.134)	0.004
Third	0.943 (0.432–2.059)	0.882	4.473 (2.145–9.319)	0.001
Fourth	3.993 (1.685–9.462)	0.002	3.435 (1.665–7.085)	0.001

Abbreviations: CL, Confidence interval; OR, odds ratio; PSCI, post-stroke cognitive impairment; SII, systemic immune-inflation. Model 1 adjusted for age, and sex; Model 2 adjusted for age, sex, education years, hypertension, diabetes, stroke subtypes, baseline NIHSS score, and Hs-CRP levels.

## Data Availability

The data that support the findings of this study are available from the corresponding author upon reasonable request.
